# Impact of Family Socioeconomic Status on Health‐Related Quality of Life in Children With Critical Congenital Heart Disease

**DOI:** 10.1161/JAHA.118.010616

**Published:** 2018-12-19

**Authors:** Li Xiang, Zhanhao Su, Yiwei Liu, Yuan Huang, Xiaoling Zhang, Shoujun Li, Hao Zhang

**Affiliations:** ^1^ State Key Laboratory of Cardiovascular Disease and Key Laboratory of Cardiac Regenerative Medicine Fuwai Hospital National Center for Cardiovascular Diseases Chinese Academy of Medical Sciences and Peking Union Medical College Beijing China; ^2^ Center for Pediatric Cardiac Surgery Fuwai Hospital National Clinical Research Center for Cardiovascular Diseases Beijing China

**Keywords:** congenital heart disease, health‐related quality of life, socioeconomic status, Quality and Outcomes, Congenital Heart Disease

## Abstract

**Background:**

Socioeconomic status (SES) is associated with health‐related quality of life (HRQOL) for children with critical congenital heart disease; however, literature from newly industrialized countries is scarce.

**Methods and Results:**

This cross‐sectional study included 2037 surviving patients operated on for critical congenital heart disease at a tertiary hospital in China between May 2012 and December 2015. All eligible patients were aged 2 to 12 years. HRQOL was measured by the Pediatric Quality of Life Inventory 4.0 generic and 3.0 cardiac modules. Family SES was assessed by a composite of household income in the past year and occupation and education level of each parent in the family. Mean scores of major domains in HRQOL were significantly lower in the low‐SES group than in the medium‐ and high‐SES groups (total generic scores: 71.2±7.9 versus 75.0±8.0 and 76.0±7.9, respectively [*P*<0.001]; psychosocial functioning: 70.8±9.0 versus 74.4±8.4 and 75.3±8.4 [*P*<0.001]; physical functioning: 71.6±10.4 versus 76.0±9.7 and 77.1±9.4 [*P*<0.001]; heart symptoms: 71.9±11.6 versus 75.7±11.0 and 76.8±10.3 [*P*<0.001]; cognitive problems: 65.4±11.1 versus 69.4±12.1 and 74.6±13.6 [*P*<0.001]). After adjustment for other clinical and demographic variables in the multivariable linear regression model, family SES significantly affected all dimensions of HRQOL except for treatment barriers, treatment anxiety, physical appearance and communication.

**Conclusions:**

Family SES is an important factor associated with HRQOL in patients with critical congenital heart disease. Further targeted interventions to improve HRQOL that consider the family and environmental issues confronted by those who are economically disadvantaged might help these patients have better outcomes.

**Clinical Perspective**

**What Is New?**

- Intergroup differences in subscales of the Pediatric Quality of Life Inventory were observed across tertiles for socioeconomic status among patients with critical congenital heart disease, except for physical appearance, treatment barriers, treatment anxiety, and communication.
- Family socioeconomic status is an independent determining factor for health‐related quality of life in patients with critical congenital heart disease.

**What Are the Clinical Implications?**

- Findings from this article will enable us to create targeted interventions that consider the family and environmental issues confronted by socioeconomically disadvantaged patients so as to improve health‐related quality of life in the growing critical congenital heart disease population.
- Incorporating family socioeconomic factors into the assessment of health‐related quality of life in pediatric patients may provide more insights to inform clinical practice and resource allocation.

## Introduction

In the field of critical congenital heart disease (CCHD), accumulating advances in surgical skills and perioperative management have reduced both morbidity and mortality in many patients and significantly improved their mid‐ and long‐term survival. CCHD has become a chronic condition, and these patients face multiple challenges such as debilitating physical symptoms, unexpected complications, and impaired psychological and social functioning,^1^, ^2^ which has negative impacts on their health‐related quality of life (HRQOL). Compared with healthy peers, patients with congenital heart disease (CHD) reported impaired quality of life,^3^, ^4^, ^5^, ^6^ and this gap might widen as disease and surgical complexity increases^7^, ^8^ and as age advances.^9^, ^10^ Accordingly, there is growing interest among clinicians worldwide to investigate the potential risk factors that could affect HRQOL of patients after congenital cardiac surgery.^11^ Optimizing HRQOL in CCHD patients has been increasingly important because of the association between poor HRQOL and adverse life outcomes.

The measurement of HRQOL, which generally includes physical, mental, and social health, provides systematic documentation of treatment efficacy and healthcare quality. It may inform clinical practice by identifying risk factors that could impair patient outcomes. Disease‐related factors such as cardiac diagnosis and surgical complexity are closely associated with CHD survival and complications. However, perioperative and disease factors are only marginally associated with HRQOL after surgery, as shown in a prospective cohort study of CCHD patients from Canada^12^ and a large survey of children with CHD from the United States.^13^ In addition, these factors are not helpful for predicting long‐term HRQOL in CCHD survivors, as demonstrated in a multicenter cohort study conducted in the United States and the United Kingdom.^14^ Consequently, potential nonclinical risk factors, such as social position, family structure and income, and community environment, are gaining attention and warrant further investigation.

Socioeconomic status (SES) is a composite measure including income, educational attainment, and employment status. SES is indicative of a patient's social position and has significant impact on cardiovascular health.^15^ SES is closely linked with cardiovascular disease risk profile and treatment outcomes, with a positive association between socioeconomic disadvantage and worse prognosis for cardiovascular disease.^16^, ^17^ As for CHD, our group previously demonstrated that socioeconomic disadvantage could compromise survival advantage after open‐heart surgery, as measured by higher postdischarge mortality and more unplanned readmission events in children from families with low SES.^18^ Despite substantial evidence of negative impact on treatment outcomes, the relationship between SES and HRQOL has not been fully explored, and the existing data show conflicting results. From 2008 to 2013, for instance, studies from the United States reported that higher SES predicted higher HRQOL scores in children and adolescents with CHD.^13^, ^19^ In contrast, other studies from Switzerland found that SES was not associated with any dimensions of HRQOL^20^ and that there was no association between SES and HRQOL in children at 4 years of age after cardiac surgery.^21^ These studies differ not only in study design and patient characteristics but also in cultural and social factors, which limit the interpretation of study results. More important, most studies were conducted in more economically developed countries with relatively good social welfare systems, which would probably mask the effect of socioeconomic disadvantage. In the meantime, studies from newly industrialized countries (NICs) are scarce, with a substantial knowledge gap regarding CHD surgical outcomes in terms of quality of life.^6^

To cover this knowledge gap and to examine the impact of SES factors on HRQOL, we sought to evaluate the association between family SES and parent/guardian proxy‐reported HRQOL of children after cardiac surgery for CCHD in a nationally representative cohort of China. We hypothesized that family socioeconomic disadvantage would be associated with impaired HRQOL in children after critical cardiac surgery in NICs.

## Methods

The data, analytic methods, and study materials will not be made available to other researchers for purposes of reproducing the results or replicating the procedure because of the privacy rules of data administration of Fuwai Hospital (Beijing, China).

### Study Design and Participants

This study is an ancillary study of a prospective observational cohort study based in Fuwai Hospital. Briefly, the prospective cohort study included 2555 patients who were operated on for CCHD in the tertiary medical center between May 2012 and December 2015. For the 2485 patients discharged alive, follow‐up was conducted regularly (1, 6, and 12 months after the date of discharge and annually thereafter) at the clinic or via telephone to track survival and health status of these patients, as described in detail previously.^18^

From November 1, 2016, to May 4, 2017, we performed the last follow‐up and successfully contacted a total of 2106 surviving subjects from the original cohort who were enrolled in this ancillary study. Patients were excluded if 1 of the following criteria were met: (1) aged <2 years at the recruitment time point of this study; (2) had noncardiac comorbidities or major neurological disabilities that could affect HRQOL; (3) had a history of heart transplantation; (4) had recent surgical procedures (<6 months).

Enrolled patients and their parents/guardians returning to Fuwai Hospital for routine outpatient follow‐up were consecutively asked to participate in this study and to complete written surveys. Patients who were not scheduled for a clinic visit during the study period were contacted by telephone. The telephone interview involved reading the instructions, questions, and all possible responses verbatim. Well‐trained research assistants collected the data and scored the Pediatric Quality of Life Inventory (PedsQL) questionnaires according to the responses of the parents/guardians. This study was approved by the hospital institutional review board and conducted according to the Declaration of Helsinki. Verbal or written informed consent was obtained from a parent or guardian of each child.

### Data Collection

As reported in our previous study,^18^ demographic characteristics (sex, date of birth, family address, number of siblings, marital status) and clinical variables (primary diagnosis of CCHD, operation types, presence of an implantable cardioverter‐defibrillator and/or pacemaker) were collected through self‐report by parents and from electronic medical records at baseline. The operation types were divided into *biventricular repair* and *other*. Details on classifications of CCHD and the last operation types are presented in Tables S1 and S2. For the purpose of this study, at the cross‐sectional time point, we updated information on number of siblings, family address (residence in urban or rural areas and residence in a lower income county), marital status, and family SES during clinic or telephone interview. Data regarding numbers of open‐heart surgeries, medication use in the past month, and hospital readmission in the past 12 months were also obtained.

To comprehensively evaluate the SES of each family, the measurement of family SES includes household income in the past year, occupation, and education level of each parent. The rationale, feasibility, and validity of adopting this method to measure family SES was extensively described in our previous report,^18^ and similar methods are described in other studies.^22^, ^23^ Briefly, annual household income was categorized as *low* (<¥10 000; score 1), *medium‐low* (¥10 000–29 999, score 2), *medium* (¥30 000–49 999, score 3), *medium‐high* (¥50 000–99 999, score 4), and *high* (≥¥100 000, score 5). For each parent, education was categorized as *less than high school* (low, score 1), *high school graduate or equivalent* (medium, score 2), and *college graduate or above* (high, score 3). Occupation included manual worker, farmer, or unemployed (low, score 1); businessman or clerk (medium, score 2); and professional, manager, or government employee (high, score 3). All the updated SES information was reported by parents and collected through a standardized form. After combining scores from these domains, we stratified patients into approximate tertiles of family SES score distribution (Figure S1): *low* (scores 5–8), *medium* (scores 9–11), and *high* (scores 12–17).

### Measurement of HRQOL

HRQOL outcomes were assessed using the PedsQL 4.0 generic core scales and 3.0 cardiac modules. The PedsQL 4.0 generic core scales is a validated multidimensional assessment tool for children aged 2 to 18 years^24^ and encompasses 23 items that assesses physical (8 items), emotional (5 items), social (5 items), and school functioning (5 items). Psychosocial health is measured as a combination of the emotional, social, and school functioning dimensions. The 3.0 cardiac module is a disease‐specific module and also has 6 subscales evaluating HRQOL related to heart problems and treatment (7 items), treatment barriers (3 items), physical appearance (3 items), treatment anxiety (4 items), cognition (5 items), and communication (3 items). The validity and reliability of this scale has been demonstrated in other studies.^13^ The Chinese Mandarin versions of the PedsQL 4.0 generic and 3.0 cardiac modules were provided by MAPI Research Institute (signed agreement for use; Lyon, France). The Chinese versions of the PedsQL generic core scales and cardiac module have been validated in Chinese children populations and showed good psychometric properties.^25^, ^26^, ^27^

Because the majority of patients (1378/2037, 67.6%) in our study were aged <5 years and most follow‐up was conducted via telephone, we adopted proxy‐report HRQOL for all patients. All scores were reported by parents/guardians. Parents/guardians answered each item for HRQOL outcomes using a 5‐point Likert scale ranging from 0 to 4 (0, never a problem; 1, almost never a problem; 2, sometimes a problem; 3, often a problem; 4, almost always a problem). If the included patients did not have any experience related to the following subscales in the past month, including schooling, treatment barriers, treatment anxiety, cognition, and communication, then the corresponding items were not scored and left blank. The scored responses are reverse scored and linearly transformed to a 0 to 100 scale (0=100, 1=75, 2=50, 3=25, 4=0), with higher scores delegating better HRQOL.

### Statistical Analyses

Continuous variables were reported as means with standard deviations or medians with interquartile ranges, depending on normality of distribution. The Shapiro–Wilk test was used to assess normality distribution of continuous variables. Categorical variables were summarized as frequencies and proportions. Continuous variables were compared across the SES tertiles using the Kruskal–Wallis test or 1‐way ANOVA, and the χ^2^ test was performed for categorical variables. We constructed multivariable linear regression models to assess the association between family SES and subscales of PedsQL scores. We forced age and sex into the multivariable models for their clinical relevance to HRQOL. Other clinical and demographic parameters with a *P* value <0.2 in univariate analysis were also entered into the models. Categorical variables with >2 categories were transformed into dummy variables.

Sociodemographic and clinical data were complete with no missing data, owing to routine follow‐up and data collection in our original cohort. In addition, because of direct contact with the parents/guardians during follow‐up, we did not have missing values in HRQOL data. All tests were 2‐sided, and *P*<0.05 was considered statistically significant. All analyses were performed using SPSS 19.0 (IBM Corp). Patients from single‐parent households were excluded in the final analysis because our composite measure of SES required information from both parents. Considering the generalizability of findings, we assigned children from single‐parent households into low and high SES, respectively, to perform sensitivity analysis.

## Results

### Characteristics of the Study Population

Figure presents the study procedure and the number of patients excluded from the original cohort. Briefly, of the 2106 surviving patients, 36 were excluded for single‐parent household and noncardiac comorbidities and neurological disabilities. Thirty‐three patients refused to participate in this study, and no patient required heart transplantation or underwent a surgical procedure within 6 months of enrollment, which leaves a total of 2037 patients in our final analysis (response rate: 96.7%, 2037/2106). Table 1 shows the demographic characteristics of the study population. The median age at cross‐sectional assessment was 4.2 years (interquartile range: 2.9–5.4 years), with an age range between 2 to 12 years. The median follow‐up time after the last operation was 33.5±13.4 months. Among these survivors, 1230 (60.4%) were boys, 1182 (58.0%) were from rural areas, and 297 (14.6%) were from lower income counties. Overall, 967 mothers (47.5%) and 913 fathers (44.8%) reported an education level of below high school, and 1283 mothers (63.0%) and 1056 fathers (51.8%) were unemployed or employed as farmer or laborer. Across socioeconomic tertiles, differences in household income, occupation and education level of each parent, and place of residence (rural/urban) were statistically significant among low‐, medium‐, and high‐SES groups (Table 1). It is noteworthy that children from low‐SES families were more likely from rural areas and lower income counties (both *P*<0.001) and have siblings (*P*<0.001) than children from medium‐ or high‐SES families (Table 1).

**Figure 1:**
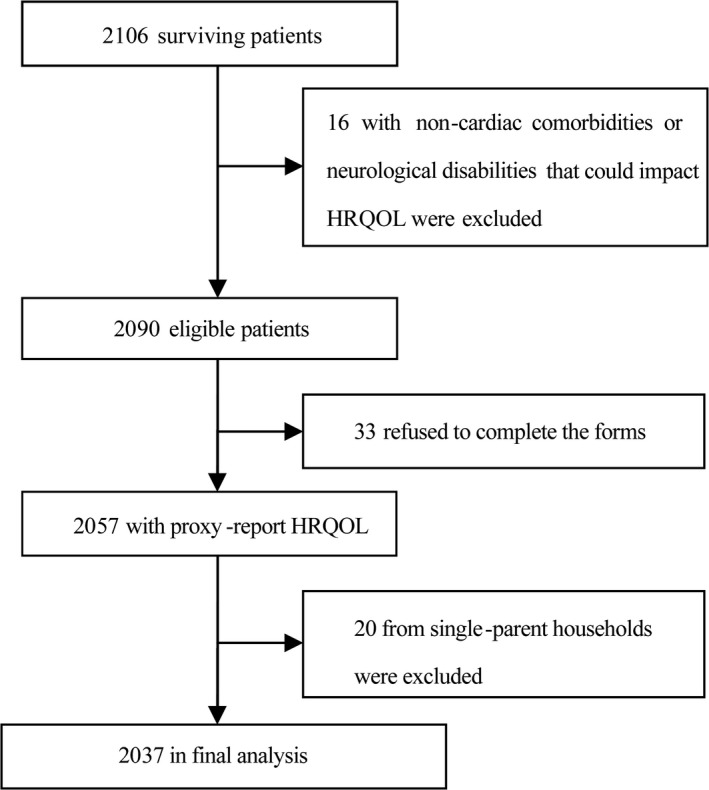
Study cohort inclusion and exclusion criteria. HRQOL indicates health‐related quality of life.

**Table 1:** Demographic Characteristics by SES Tertiles

Characteristic	SES			*P* Value^a^
	Low (n=723)	Medium (n=678)	High (n=636)	
Age at PedsQL completion, y, median (IQR)	4.3 (3.0–5.5)	4.2 (2.9–5.4)	3.9 (2.7–5.1)	0.001
Age range, y				0.007
2–4	467 (64.6)	448 (66.1)	463 (72.8)	
5–7	206 (28.5)	180 (26.5)	147 (23.1)	
8–12	50 (6.9)	50 (7.4)	26 (4.1)	
Sex				0.24
Male	443 (61.3)	420 (61.9)	367 (57.7)	
Female	280 (38.7)	258 (38.1)	269 (42.3)	
Paternal education				<0.001
Low	661 (91.4)	231 (34.1)	21 (3.3)	
Medium	62 (8.6)	415 (61.2)	252 (39.6)	
High	0 (0)	32 (4.7)	363 (57.1)	
Maternal education				<0.001
Low	673 (93.1)	269 (39.7)	25 (3.9)	
Medium	50 (6.9)	395 (58.3)	284 (44.7)	
High	0 (0)	14 (2.1)	327 (51.4)	
Paternal occupation				<0.001
Low	679 (93.9)	345 (50.9)	32 (5.0)	
Medium	43 (5.9)	293 (43.2)	371 (58.3)	
High	1 (0.1)	40 (5.9)	233 (36.6)	
Maternal occupation				<0.001
Low	699 (96.7)	473 (69.8)	111 (17.5)	
Medium	24 (3.3)	184 (27.1)	332 (52.2)	
High	0 (0)	21 (3.1)	193 (30.3)	
Annual household income				<0.001
Low	29 (4.0)	4 (0.6)	1 (0.2)	
Medium‐low	226 (31.3)	55 (8.1)	18 (2.8)	
Medium	308 (42.6)	217 (32.0)	65 (10.2)	
Medium‐high	160 (22.1)	293 (43.2)	280 (44.0)	
High	0 (0)	109 (16.1)	272 (42.8)	
Rural residence	605 (83.7)	387 (57.1)	190 (29.9)	<0.001
Residence in a lower income county	156 (21.6)	91 (13.4)	50 (7.9)	<0.001
Presence of sibling				<0.001
Yes	456 (63.1)	355 (52.4)	290 (45.6)	
No	267 (36.9)	323 (47.6)	346 (54.4)	

Values are n (%) except as noted. Parental occupation was divided into manual worker, farmer, or unemployed (low); businessman or clerk (medium); and professional, manager, or government employee (high). Annual household income was categorized as low (<¥10 000), medium‐low (¥10 000–29 999), medium (¥30 000–49 999), medium‐high (¥50 000–99 999), and high (≥¥100 000). Parental education level was categorized as less than high school (low), high school graduate or equivalent (medium), and college graduate or above (high). IQR indicates interquartile range; PedsQL, Pediatric Quality of Life Inventory; SES, socioeconomic status.

a *P* value for comparison of SES groups using the χ^2^ or Kruskal–Wallis test.

Table 2 describes the clinical features of the study population. Patients with low SES had a lower proportion of biventricular repair (*P*<0.001) and a higher proportion of other procedures (*P*<0.001) than patients with medium and high SES, which likely indicates delayed intervention at their index operation, as described in our previous report.^18^ Notably, patients from low‐SES families, compared with medium‐ and high‐SES families, had a significantly higher proportion of medication use in the past month (46.6% versus 42.2% and 39.0%, respectively; *P*=0.02) and a higher percentage of hospital admission in the past 12 months (18.4% versus 16.8% and 11.3%, respectively; *P*=0.001). Among the 3 groups, there are marginal differences with respect to the presence of an implantable cardioverter‐defibrillator and/or pacemaker and history of multiple open‐heart surgeries.

**Table 2:** Clinical Features of Study Population by SES Tertiles

Characteristic	SES			*P* Value^a^
	Low (n=723)	Medium (n=678)	High (n=636)	
Operation type				<0.001
Biventricular repair	564 (78.0)	549 (81.0)	554 (87.1)	
Other	159 (22.0)	129 (19.0)	82 (12.9)	
Presence of ICD and/or pacemaker^b^				0.99
Yes	3 (0.4)	3 (0.4)	3 (0.5)	
No	720 (99.6)	675 (99.6)	633 (99.5)	
History of multiple open‐heart surgeries				0.16
Yes	91 (12.6)	87 (12.8)	62 (9.7)	
No	632 (87.4)	591 (87.2)	574 (90.3)	
Medication use in the past month				0.02
Yes	337 (46.6)	286 (42.2)	248 (39.0)	
No	386 (53.4)	392 (57.8)	388 (61.0)	
Past 12‐mo hospital admission				0.001
Yes	133 (18.4)	114 (16.8)	72 (11.3)	
No	590 (81.6)	564 (83.2)	564 (88.7)	
Follow‐up period since last operation, mo, mean (SD)	33.6 (13.1)	34.7 (13.7)	34.3 (13.4)	0.31

Values are n (%) except as noted. ICD indicates implantable cardioverter‐defibrillator; SES, socioeconomic status.

a *P* value for comparison of SES groups using the χ^2^ test or 1‐way ANOVA.

b Corrected χ^2^ test.

### Comparisons of PedsQL Scores by Socioeconomic Tertiles

On the PedsQL generic core scales, patients from low‐SES families had significantly lower PedsQL scores in all domains than patients with medium or high SES (all *P*<0.001; Table 3). On the PedsQL Cardiac Module, mean heart problems and treatment and cognitive problems scale scores were significantly lower for children from low‐SES families than from medium‐ or high‐SES families (all *P*<0.001). There were no statistically significant differences in parent/guardian‐reported scores for treatment barriers, treatment anxiety, physical appearance, and communication for pediatric patients with CCHD across different SES groups.

**Table 3:** Comparisons of PedsQL Scores by SES Tertiles

	PedsQL Score						*P* Value^a^
	Low SES		Medium SES		High SES		
	n	Mean (SD)	n	Mean (SD)	n	Mean (SD)	
Generic scale							
Proxy report							
Total score	723	71.2 (7.9)	678	75.0 (8.0)	636	76.0 (7.9)	<0.001
Psychosocial functioning	723	70.8 (9.0)	678	74.4 (8.4)	636	75.3 (8.4)	<0.001
Physical functioning	723	71.6 (10.4)	678	76.0 (9.7)	636	77.1 (9.4)	<0.001
Emotional functioning	723	68.4 (11.3)	678	72.5 (10.2)	636	72.6 (9.9)	<0.001
Social functioning	723	72.1 (11.9)	678	76.3 (11.5)	636	77.2 (11.4)	<0.001
School functioning	422	74.5 (11.4)	412	75.3 (11.0)	355	79.1 (11.1)	<0.001
Cardiac scale							
Proxy report							
Heart problems and treatment	723	71.9 (11.6)	678	75.7 (11.0)	636	76.8 (10.3)	<0.001
Treatment barriers	70	94.3 (6.8)	66	94.0 (7.2)	43	93.2 (7.1)	0.74
Physical appearance	723	90.2 (12.6)	678	90.0 (12.6)	636	91.4 (11.3)	0.06
Treatment anxiety	298	83.5 (14.4)	248	84.4 (12.9)	221	84.0 (13.5)	0.72
Cognitive problems	710	65.4 (11.1)	673	69.4 (12.1)	630	74.6 (13.6)	<0.001
Communication	298	75.8 (12.0)	248	77.0 (11.6)	221	75.0 (11.6)	0.19

PedsQL indicates Pediatric Quality of Life Inventory; SES, socioeconomic status.

a *P* value for comparison of SES groups using 1‐way ANOVA.

### Multivariable Analysis of Family SES and HRQOL for Patients With CCHD

Subscales of HRQOL that were significantly associated with family SES (*P*<0.20) in univariate analysis are shown in Table S3. After adjustment for demographic and clinical variables, multivariable analysis shows strong associations between family SES and PedsQL generic scores (Table 4). There were statistically significant differences with regard to total generic score, psychosocial functioning, physical functioning, social and emotional functioning (high versus low SES, all *P*<0.001; medium versus low SES, all *P*<0.001). As for school functioning, the difference was significant for high‐ versus low‐SES groups (*P*<0.001) but not for medium‐ versus low‐SES groups (*P*=0.24).

**Table 4:** Tests of Multivariable Linear Regression Analysis of PedsQL Scores With SES

PedsQL Scale	SES (High vs Low)						SES (Medium vs Low)						Adj *R* ^2^
	B	95% CI	SEB	β	*t* Value	*P* Value	Β	95% CI	SEB	β	*t* Value	*P* Value	
Total generic score	3.72	2.85–4.59	0.44	0.21	8.40	<0.001	3.42	2.64–4.20	0.40	0.20	8.60	<0.001	0.24
Psychosocial functioning	3.54	2.57–4.51	0.49	0.19	7.18	<0.001	3.20	2.33–4.07	0.44	0.17	7.23	<0.001	0.18
Physical functioning	3.92	2.81–5.03	0.57	0.18	6.94	<0.001	3.71	2.71–4.70	0.51	0.17	7.30	<0.001	0.19
Emotional functioning	3.89	2.65–5.14	0.64	0.17	6.13	<0.001	3.95	2.83–5.07	0.57	0.17	6.90	<0.001	0.17
Social functioning	3.80	2.51–5.09	0.66	0.15	5.78	<0.001	3.48	2.33–4.64	0.59	0.14	5.90	<0.001	0.18
School functioning	3.70	2.04–5.35	0.84	0.15	4.38	<0.001	0.84	−0.57–2.25	0.72	0.04	1.17	0.24	0.21
Heart problems and treatment	2.93	1.79–4.07	0.58	0.12	5.04	<0.001	2.94	1.92–3.96	0.52	0.12	5.63	<0.001	0.29
Cognitive problems	8.30	6.84–9.76	0.75	0.30	11.1	<0.001	3.77	2.46–5.08	0.67	0.14	5.64	<0.001	0.12
Physical appearance	0.38	−0.99–1.74	0.70	0.01	0.54	0.59	−0.31	−1.54–0.92	0.63	−0.01	−0.50	0.62	0.14

Factors that reached a *P* value <0.2 in univariate analysis were included in the multiple regression model. Age and sex were included in the model. Factors are common for all subscales including SES (high vs low; medium vs low), age (8–12 vs 2–4 y; 5–7 vs 2–4 y), sex (male vs female), rural residence (no vs yes), operation type (biventricular repair vs other), history of multiple open‐heart surgeries (no vs yes), past 12‐mo hospitalization; follow‐up period since last operation. Medication use in the past month (no vs yes) is for all subscales with the exception of physical appearance. Presence of ICD and/or pacemaker (no vs yes) is only for social functioning and heart problems and treatment. Presence of sibling (yes vs no) is only for total score, physical functioning, social functioning, and heart problems and treatment. Residence in a lower income county (no vs yes) is for all subscales except physical appearance and emotional functioning. CI indicates confidence interval; ICD, implantable cardioverter‐defibrillator; PedsQL, Pediatric Quality of Life Inventory; SEB, standard error of the regression coefficient; SES, socioeconomic status.

The multivariable analysis also demonstrates strong associations between family SES and subscales of PedsQL cardiac module. Low family SES had significant impacts on heart problems and treatment and cognitive problems (both *P*<0.001) compared with medium and high family SES. However, the differences were not significant in physical appearance (high versus low SES: *P*=0.59; medium versus low SES: *P*=0.62). In the regression model, the variability in HRQOL scales explained by the independent variables ranges from 12% to 24%. For all influenced subscales, SES accounted for 20% to 66% of the total *R* ^2^ in HRQOL dimensions.

In sensitivity analyses that assigned all 20 patients from single‐parent households into low‐ or high‐SES groups, there were no significant changes in the main clinical findings (data not shown).

## Discussion

This large cross‐sectional study from China demonstrated an inverse relationship between family SES and HRQOL for pediatric patients after CCHD surgery. Compared with children from families with the lowest SES, those from medium‐ and high‐SES families reported higher scores across almost all dimensions of HRQOL, indicating better physical and psychosocial health. These findings persisted in analyses after adjustment for several demographic and clinical variables in the multivariable linear regression models. Our results are statistically significant, but it is reasonable to question whether these results were clinically meaningful. A prior study^28^ suggests that the minimally important difference on HRQOL measures is about 0.5 SD, which is derived from 33 different studies including various disease conditions. However, no definition of clinically significant difference yet exists for the PedsQL measure. Using the same PedsQL scales, previous studies^13^, ^22^ reported that the minimum difference of means was 3.07 (*P*<0.05) between a healthy sample and patients with mild cardiovascular disease and 3.7 (*P*<0.05) between a healthy sample and Fontan patients. It is generally accepted that there are significant differences in HRQOL scores between Fontan patients and healthy participants. In our study, the mean difference in HRQOL across SES tertiles ranges from 3.6 to 9.2. We perceived that the difference in HRQOL between the low‐SES group and those with medium and high SES might be clinically significant. Longer follow‐up in the future is needed to verify this conclusion.

In this study, patients came from regions all across China with tremendous geographic and cultural diversity as well as unbalanced economic development. The measurement of family SES in our study, which takes into account household income levels, occupation status, and education attainment, is suitable for the cultural and political–economic context of China. Many studies have described the critical role of SES in mid‐ and long‐term quality of life for patients with congenital heart defects. In a multicenter study from the United States, multiple family SES variables were found to be correlated with HRQOL in CHD patients, with family income having the strongest impact on HRQOL outcomes.^18^ Two other single‐center studies from North America also identified higher SES as being associated with better quality of life after pediatric cardiac surgery.^12^, ^13^ Our findings are consistent with these previous studies, which demonstrated a clear link between SES and HRQOL in CCHD patients. Furthermore, our study adds to the limited literature that directly investigates SES and HRQOL in CHD. To our best knowledge, this research is the first and the largest cross‐sectional study from an NIC to report such findings.

In the present study, operation types (biventricular repair versus other) are predictive for almost all subscales of HRQOL. However, surgical complexity might not be associated with HRQOL in CHD patients, as shown in previous studies.^12^, ^14^ This inconsistent finding could be explained by difference in follow‐up interval and patient population, as well as support network in the community. The increasing burden of cardiac‐ and non–cardiac‐related medical interventions over the lifetime has a significantly negative impact on patient HRQOL.^4^ In our study, patients from lower SES families were more likely to have used medication in the past month and to have been admitted to the hospital in the past 12 months, and these low‐SES patients had lower scores on HRQOL domains. This phenomenon can be explained by several factors related to family SES. First, postdischarge care is crucial to CHD patient survival and functional recovery, which is intimately related with parental support and family environment.^29^ Going home with a pediatric patient after a cardiac procedure poses major challenges for many parents,^30^ and it is foreseeable that parents who have lower educational backgrounds and who are financially disadvantaged would face more pressure in handling this situation. Second, taking good care of sick children requires certain levels of health knowledge, especially for those with special healthcare needs such as CHD.^31^ Parents from low‐SES families with low education levels tended to have lower health literacy, which would probably lead to more unintended hospital admissions and higher utilization of resources at local health facilities. In this regard, our results are consistent with previous findings,^32^ and low health literacy in parents is closely correlated with poor child health outcomes.^33^ Consequently, children from lower SES families would probably have more health problems and admission events due to low parental health literacy, and this might severely impair their HRQOL. Moreover, patients from lower SES families were more likely from rural areas or lower income counties, which could hinder their access to care at tertiary medical centers in metropolitan regions. Potential measures including telemedicine in rural regions could create a bridge between experienced doctors and patients. Telemedicine might solve the issue of access to care and benefit rural patients.

Physical health is a key domain of HRQOL to assess the functional recovery status of patients. Even though surgical treatment for critical heart defects improves physical ability to some extent, chronic disease status still limits patient HRQOL. However, disease itself might not affect self‐perceived physical health of CCHD patients because HRQOL is more strongly associated with perceptions of illness and functional status rather than defect severity.^34^ Our results show that CCHD patients from lower SES families had significantly lower scores for physical health, which indicates impaired physical functioning in these patients. This is consistent with the findings from a large cross‐sectional study of Fontan patients that revealed lower family income is significantly associated with worse physical functioning.^7^ Similarly, another report from the United Kingdom showed that adult CHD patients living in socioeconomically deprived communities had lower exercise tolerance and physical ability,^35^ an effect likely mediated through reduced access to physical activity resources and more air pollution in these areas. As such, socioeconomic disadvantage deprives CCHD patients of optimal physical recovery and negatively affects their HRQOL independent of the underlying disease.

Likewise, the relationship between cyanotic CHD and poorer neurodevelopmental outcomes has been well documented in much of the literature.^36^, ^37^, ^38^, ^39^ Concomitantly, CHD patients had impaired cognitive function and worse academic performance compared with healthy peers.^37^ Our study found that patients from low‐SES families had more cognitive problems and poorer school functioning than patients from high‐SES families. This finding is in line with earlier studies showing that low SES is associated with impaired neurocognitive development^40^, ^41^, ^42^ and poor academic performance in school‐aged children.^43^ Difficulties in academic study and school performance among patients with CCHD would bring psychological, emotional, and social problems,^44^ which further compromise the HRQOL of patients from lower SES families.

Measurement of HRQOL for patients across different countries poses a challenge to integrating the results of different study findings. Indeed, some studies from more economically developed countries like Switzerland^20^, ^21^ found that SES is not correlated with any dimensions of HRQOL of CHD patients. This discrepancy could be partially explained by inconsistent methodology and cultural differences between countries. However, a recent global report of HRQOL of adult CHD patients found that country‐specific characteristics such as national happiness level were not associated with HRQOL variation and stressed that CHD patients at risk for impaired HRQOL can be identified using the same criteria.^11^ Other country‐specific factors, like health care and social welfare system, might very likely mediate these country‐specific effects. As a result, study findings from more economically developed countries might not be applicable to patients from NICs. In view of this information, our study adds to the very limited literature that assesses the effect of SES on HRQOL of CCHD patients from a NIC setting.

This study has several limitations. First, potential selection bias exists because of higher postdischarge mortality in low‐SES patients. Second, exclusion of patients with noncardiac comorbidities or neurological disability might have led to better reported scores than those of the actual CCHD population. Third, we identified 20 single‐parent families during the study period and could not obtain complete SES data from them. Family structure might be associated with the child's HRQOL, and single‐parent household status might have an effect on patient prognosis.^45^ In our cross‐sectional study, children from single‐parent households were excluded, but the number of excluded patients is small and did not alter our results. Moreover, family SES changes over time. Based on the number of patients and their updated SES scores, we divided our patients into 3 groups according to SES tertile. The grouping cutoff values differ slightly from our previous study, reflecting changes in SES over time, and we did not identify the specific component of SES that had the strongest effect on prognosis. Fourth, the evaluation of HRQOL was reported by proxy. Several studies have reported variability between the proxy and child reports of HRQOL.^46^ However, some studies found that proxy and child reports of HRQOL agreement are high for young children but low for teenagers.^5^ Because most patients in our study were <5 years old, we adopted only proxy‐reported measurement of HRQOL. Finally, for all influenced HRQOL subscales, regression models explained <30% of the variability in parental reports, indicating that other important variables may not be included in this study.

In conclusion, we found that after surgical treatment for CCHD, patients from low‐SES families had worse physical, psychosocial, and school functioning than patients from higher SES families. Moreover, family SES is an independent risk factor for HRQOL after adjustment for other clinical and demographic variables. For clinicians, HRQOL should be included in routine assessment of health status for pediatric patients after surgical treatment of CHD, particularly for those with significant socioeconomic disadvantage. Moreover, findings from this study will enable us to create targeted interventions that take into consideration family and environmental issues confronted by those who are economically disadvantaged so as to improve the HRQOL of the CCHD patient population.

## Sources of Funding

The study was funded by the Program for Professors at Peking Union Medical College (to H. Zhang), the National Science Fund for Distinguished Young Scholars (81525002; to H. Zhang), and the National Key R&D Program of China (2017YFC1308100; to S. Li and H. Zhang).

## Disclosures

None.

## Supporting information


**Table S1.** Details on Classifications of Critical Congenital Heart Disease
**Table S2.** Details on Operation Type of Study Population by Socioeconomic Status Tertile
**Table S3.** Univariate Analysis of Factors Associated With Pediatric Quality of Life Inventory Dimensions at Cross‐Sectional Assessment
**Figure S1.** Distribution of socioeconomic score.
