# Supplementary material for: Impact of Family Socioeconomic Status on Health‐Related Quality of Life in Children With Critical Congenital Heart Disease
Source: J Am Heart Assoc. 2018 Dec 19;8(1):e010616. doi: 10.1161/JAHA.118.010616 (PMC6405710; doi:10.1161/JAHA.118.010616)

Supplemental Material

Table S1. Details on classifications of CCHD.

**Primary diagnosis**	**Number (%)**
Tetralogy of Fallot	**908(44.6)**
Double outlet right ventricle	**246(12.1)**
Pulmonary atresia	**202(9.9)**
Transposition of great arteries	**189(9.3)**
Single ventricle	**130(6.4)**
Atrioventricular septal defect	**120(5.9)**
Total anomalous pulmonary vein drainage	**124(6.1)**
**Miscellaneous**	**118(5.8)**
truncus arteriosus communis	10
Ebstein anomaly	30
interrupted or hypoplastic aortic arch	18
congenitally corrected transportation of great arteries	60

Table S2. Details on operation type of study population by socioeconomic status tertile.

Operation Type	N=2037
**Biventricular repair**	**1667(81.8%)**
**Others**	**370(18.2%)**
Pulmonary artery banding	19
Modified Blalock Taussig shunt	52
Sano shunt/right ventricle-pulmonary artery shunt	15
Brock procedure	10
Bidirectional cavopulmonary shunt	143
Total cavopulmonary connection	99
One and a half ventricle repair	32

Table S3. Univariate analysis of factors associated with PedsQL dimensions at cross-sectional assessment.

	Total	Psychosocial health	Physical health	Emotional functioning	Social functioning	School functioning	Symptoms	Physical appearance	Cognitive problems
SES (high vs low)	<0.001	<0.001	<0.001	<0.001	<0.001	<0.001	<0.001	0.05	<0.001
SES (middle vs low)	<0.001	<0.001	<0.001	<0.001	<0.001	0.34	<0.001	0.80	<0.001
Age (5-7 vs 2-4)	<0.001	0.004	0.04	0.95	<0.001	<0.001	0.03	<0.001	0.25
Age (8-12 vs 2-4)	0.002	<0.001	<0.001	0.03	<0.001	<0.001	0.001	<0.001	<0.001
Sex (female vs male)	0.44	0.41	0.59	0.44	0.78	0.63	0.15	0.01	0.89
Rural residence (no vs yes)	<0.001	<0.001	<0.001	0.008	<0.001	0.12	<0.001	0.062	<0.001
Residence in a Lower income counties (no vs yes)	<0.001	0.006	<0.001	0.22	0.002	0.10	0.001	0.99	0.04
Presence of sibling (yes vs no)	0.12	0.41	0.06	0.78	0.14	0.96	0.08	0.62	0.32
Operation type (BR vs Others)	<0.001	<0.001	<0.001	<0.001	<0.001	<0.001	<0.001	<0.001	<0.001
Presence of ICD and/or pacemaker (no vs yes)	0.29	0.43	0.33	0.76	0.15	0.98	0.16	0.53	0.51
History of multiple open-heart surgeries (no vs yes)	<0.001	<0.001	<0.001	0.004	<0.001	<0.001	<0.001	<0.001	<0.001
Medication use in the past month (no vs yes)	<0.001	<0.001	<0.001	0.07	<0.001	<0.001	<0.001	0.33	0.04
Last 12 months hospital admission (no vs yes)	<0.001	<0.001	<0.001	<0.001	<0.001	<0.001	<0.001	0.03	0.07
Follow-up period since last operation (months)	<0.001	<0.001	<0.001	<0.001	<0.001	0.003	<0.001	<0.001	0.005

SES: socioeconomic status;BR: Biventricular repair

Figure S1. Distribution of socioeconomic score.

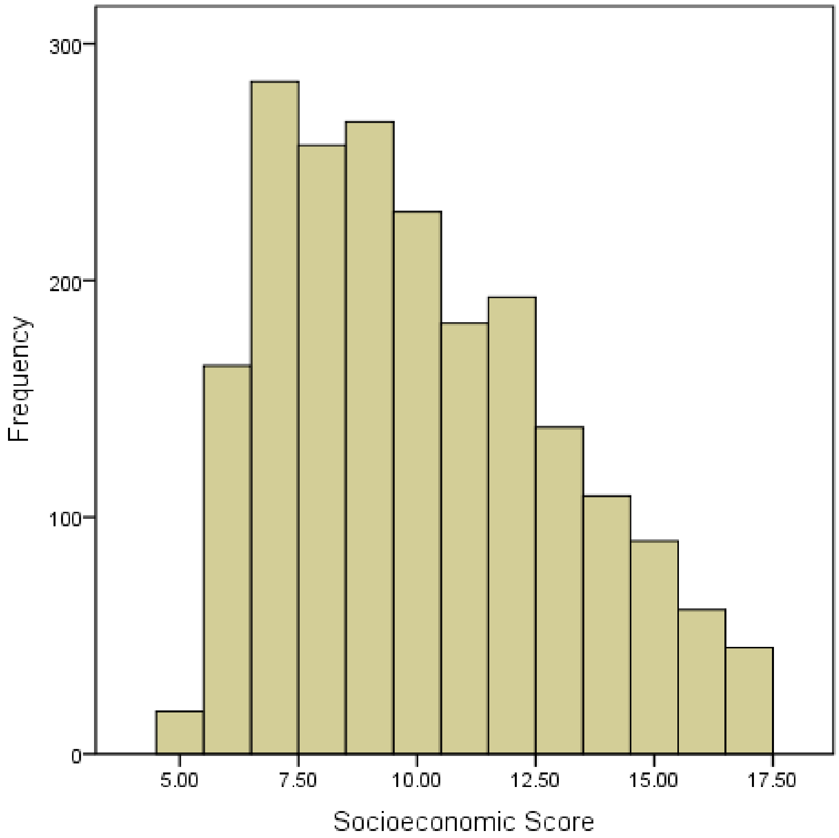

Supplement: Supplementary file 1 — Table S1. Details on Classifications of Critical Congenital Heart Disease Table S2. Details on Operation Type of Study Population by Socioeconomic Status Tertile Table S3. Univariate Analysis of Factors Associated With Pediatric Quality of Life Inventory Dimensions at Cross‐Sectional Assessment Figure S1. Distribution of socioeconomic score. [file JAH3-8-e010616-s001.pdf]
